# Examination of Molecular Effects of *MYLK* Deletion in a Patient with Extensive Aortic, Carotid, and Abdominal Dissections That Underlie the Genetic Dysfunction

**DOI:** 10.1155/2020/5108052

**Published:** 2020-06-22

**Authors:** Sarah K. Macklin, Katelyn A. Bruno, Charitha Vadlamudi, Haytham Helmi, Ayesha Samreen, Ahmed N. Mohammad, Stephanie Hines, Paldeep S. Atwal, Thomas R. Caulfield

**Affiliations:** ^1^Department of Clinical Genomics, Mayo Clinic, Jacksonville, FL 32224, USA; ^2^Research Administration, Mayo Clinic, Jacksonville, FL 32224, USA; ^3^Department of Internal Medicine & Department of Cardiovascular Diseases, Mayo Clinic, Jacksonville, FL 32224, USA; ^4^Department of Transplant and Critical Care, Mayo Clinic, Jacksonville, FL 32224, USA; ^5^Department of Neurology, Mayo Clinic, Jacksonville, FL 32224, USA; ^6^Department of Neuroscience, Mayo Clinic, Jacksonville, FL 32224, USA; ^7^Department of Cancer Biology, Mayo Clinic, Jacksonville, FL 32224, USA; ^8^Department of Health Sciences Research, Mayo Clinic, Jacksonville, FL 32224, USA

## Abstract

We describe the phenotype of a patient with extensive aortic, carotid, and abdominal dissections. The proband was found to have a heterozygous deletion of exons 21–34 in *MYLK*, which is a rare finding, as deletions in this gene have been infrequently reported. We describe this finding following detection in a proband with an extensive history of aortic, carotid, and abdominal dissections. Neoteric molecular modeling techniques to help determine the impact of this deletion on protein function indicated loss of function due to lack of any kinase domain. We also provide the electrostatics calculations from the wild type and mutant variant. Through a combined multiomic approach of clinical, functional, and protein informatics, we arrive at a data fusion for determination of pathogenicity embedded within the genetic code for this particular genetic variant, which, as a platform, continues to broaden its scope across the field of variants of uncertain significance classification.

## 1. Introduction

Thoracic aortic aneurysms leading to acute aortic dissections are a common cause of sudden death and are associated with high degree of morbidity and mortality [1]. Thoracic aortic aneurysm and dissection (TAAD) is a common feature in genetic syndromes such as Marfan syndrome, Loeys–Dietz syndrome, and certain types of Ehlers–Danlos syndrome. Even though most patients with TAAD do not have a genetic syndrome, many of them have a family history and a genetic susceptibility for aneurysms [1]. Approximately 20% of patients with nonsyndromic TAAD have a family history of the disease, and the disorder generally appears to be inherited in an autosomal-dominant manner [2]. Decreased penetrance and variable expressivity can complicate familial presentation [2].

Pathogenic variants in genes including small muscle cell contractile proteins have been implicated as causes of familial TAAD [2, 3]. Smooth muscle cells (SMCs) are a major component of the blood vessel wall, and they play an integral role in regulating blood flow and pulse pressure. The thick middle layer of the aorta, the tunica media, is composed of SMCs and elastic fibers providing the aorta with tensile strength and elasticity. Disorders that damage the structure and/or function of the elastic fibers, such as the various connective tissue syndromes, can affect the integrity of the aortic wall increasing the risk for aneurysm and subsequently dissection [1, 4].

SMCs use cross-bridge cycling between actin and myosin; however, the contractile mechanism is different from other types of muscles. The concentration of intracellular calcium (Ca^+2^) increases in SMCs when stimulated by mechanical, humoral, or electrical factors. Ca^2+^ then binds to calmodulin. This interaction is required to initiate the contraction in smooth muscles. The Ca^2+^-calmodulin complex binds to myosin light chain kinase (MYCK (OMIM #600922)) to activate it. MYCK, encoded by *MYLK*, is responsible for the phosphorylation of the 20 kDa regulatory light chain of myosin and plays a fundamental role in the activation and regulation of the contractile activity in SMCs. The myosin light chain, when phosphorylated, becomes active and increases the actin-activated myosin II ATPase activity. The energy released from the hydrolysis of ATP is required for the cross-bridge cycling of the *β*-myosin heavy chain (encoded by *MYH11*) with *α*-actin (encoded by *ACTA2*), which causes the contraction of the SMC [3–5].

In this report, a heterozygous deletion of exons 21–34 in *MYLK* is described following detection in a proband with an extensive history of aortic, carotid, and abdominal dissections. Detailed personalized structural modeling and molecular dynamic simulations were performed to help determine the impact of this deletion on protein function.

## 2. Case Presentation

The proband, a 52-year-old Caucasian male with mild hypertension, presented to the hospital after his wife found him unconscious in his garage. He recalled doing some gardening work when he felt lightheaded before losing consciousness. The patient reported that he did not experience chest pain; however, he had a sense of foreboding before he collapsed. On physical examination, he was found to have a right-sided weakness consistent with a possible stroke; he was intubated and taken immediately for CT and MR of the head and neck. Imaging studies revealed a widespread dissection of the ascending portion of the thoracic aorta with extension into the common carotid arteries and left subclavian artery accompanied by high-grade stenosis bilaterally (secondary to the dissection). The patient was also found to have a dissection of the abdominal aorta, which extended into the iliac arteries, with apparent sparing of the renal arteries.

Emergently, he underwent repair of the dissection of the ascending aorta. He had a hemiarch replacement, as well as a graft to the right axillary artery, an aortic valve repair, and valvuloplasty. Four days postoperatively, the patient developed acute left-sided hemiparesis. MRI of the brain showed predominantly right-sided cerebral hemispheric watershed infarcts with no evidence of hemorrhage. Additionally, it was found that he had a continued narrowing of the lumen of the common carotid arteries due to dissections. Because of the fragility of the vessels and the acuteness of the situation, vascular or neuroradiology intervention was not performed, and the patient was put under observation instead.

The proband underwent extensive physical therapy and rehabilitation and remarkably has recovered full physical abilities. In the course of his evaluation, he was found to have positive ANA titers up to 1 : 640. He had a homogeneous as well as a speckled pattern. He consulted with his local rheumatologist who performed an extensive serologic workup, all of which returned normal. The patient did not have any symptoms of inflammatory arthritis, rash, oral ulcers, alopecia, photosensitivity, Raynaud's phenomenon, skin thickening, skin hardening or tightening, dysphagia, cough, shortness of breath, or symptoms of serositis.

The family history does not include any known history of sudden vascular death, although his father did pass away at age 82 of a cerebral aneurysm. The patient did not display any traits of hypermobility and denies the history of recurrent joint dislocations, poor healing of the skin, or lens dislocations. He was on low-dose lisinopril for his hypertension, and he had taken prednisone long-term for his microscopic colitis, with doses of up to 30 mg per day, but usually not more than 10 mg per day.

Heritable disorders of connective tissue (HDCT) sequencing and deletion/duplication panel was performed by a CAP-accredited/CLIA-certified laboratory to explore the possibility of a vascular form of a connective tissue disorder. This test analyzed the following genes via NextGen sequencing on an Illumina platform with concurrent exon-level oligo array CGH: *ACTA2, ADAMTS2, ALDH18A1, ATP6V0A2, ATP7A, B3GALT6, B4GALT7, CBS, CHST14, COL11A1, COL11A2, COL1A1, COL1A2, COL2A1, COL3A1, COL5A1, COL5A2, COL9A1, COL9A2, DSE, EFEMP2, ELN, FBLN5, FBN1, FBN2, FKBP14, FLNA, LTBP4, MAT2A, MED12, MFAP5, MYH11, MYLK, NOTCH1, PLOD1, PRDM5, PRKG1, PYCR1, RIN2, SKI, SLC2A10, SLC39A13, SMAD3, SMAD4, TGFB2, TGFB3, TGFBR1, TGFBR2,* and *ZNF469* [UCSC hg19]. The test identified a heterozygous deletion of at least exons 21–34 of *MYLK* [3q21.1(123,332,644–123,386,568)x1 arr[GRCh37]]. The extent of the deletion was confirmed with whole-genome oligonucleotide array-based comparative genomic hybridization with single-nucleotide polymorphism analysis (CGH + SNP), arr[GRCh37] 3q21.1(123219342_123386568)x1.

All of our proband's living first-degree family members, mother, brother, and three daughters, were screened for the *MYLK* deletion. His 87-year-old mother was found to carry the same variant but had no history of aortic disease. She had completed cardiac evaluation due to the history of a myocardial infarction and placement of a pacemaker. The proband's daughter and brother also tested positive; they appear to be currently unaffected as well. His brother had completed recent cardiac imaging, which was negative. A child of the proband's brother plans to undergo further testing to see whether they carry the mutation.

### 2.1. Computer-Based Structural Modeling

The sequence of human, smooth muscle myosin light chain kinase (MYLK) (isoform 1) is a part of the muscle-contraction process that occurs through phosphorylation on myosin light chains. MYLK, which is a protein encoded by the *MYLK* gene, was taken from the NCBI Reference Accession Sequence: NP_444253: version NP_444253.3, which is encoded for the amino acid sequence and was used for computer-assisted modeling. Monte Carlo simulations were performed on the mutant to allow local regional changes for full-length 1914 amino acids and when the p.S1218del variant was introduced.

The refinement module for Monte Carlo was built using the in-house code (and YASARA) SSP/PSSM method [6–11]. The structure was relaxed to the Amber force field using knowledge-based potentials under the defined algorithms. The side chains and rotamers were adjusted with knowledge-based potentials, simulated annealing with the explicit solvent, and small equilibration simulations as described [12]. The entire full-length protein sequence was used for the structural models, and any gaps or unresolved portions from the X-ray data were filled in using our established methodology [13–21].

Refinement of the finalized models was completed using either Schrodinger's MCMD search with the Monte Carlo-based module or NAMD2 protocols. Molecular refinements started with the in-house code (built on YASARA) generated initial refinement and mutant p.S1218del [6–8, 10]. The superposition and subsequent refinement of the overlapping regions yield a complete model for MYLK. The final structures were subjected to energy optimization with the PR conjugate gradient with an *R*-dependent dielectric.

Atom consistency was checked for all 1914 amino acids (29,429 atoms) of the full-length wild-type model and 1217 amino acids (18,546 atoms) for the p.S1218del variant, verifying correctness of chain name, dihedrals, angles, torsions, nonbonds, electrostatics, atom-typing, and parameters. Each model was exported to the following formats: Maestro (MAE) and in-house code (PDB). Model manipulation was done with Maestro (Macromodel, version 9.8, Schrodinger, LLC, New York, NY, 2010) or visual molecular dynamics (VMD) [22].

Monte Carlo dynamics searching (LCMOD-MC) was completed on each model for conformational sampling using methods previously described in the literature [15, 16, 23, 24]. In brief, each system was minimized with relaxed restraints using either steepest descent or conjugate gradient PR and then allowed to undergo the MC search criteria, as shown in the literature [15, 16, 23, 24].

### 2.2. Results from Structure-Function Studies

**Figure 1:**
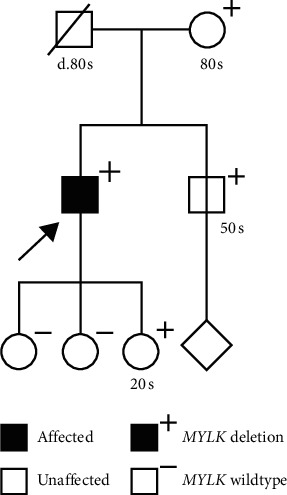
Pedigree of the proband's family. Known results from family member *MYLK*. Testing and current clinical status are included.

**Figure 2:**
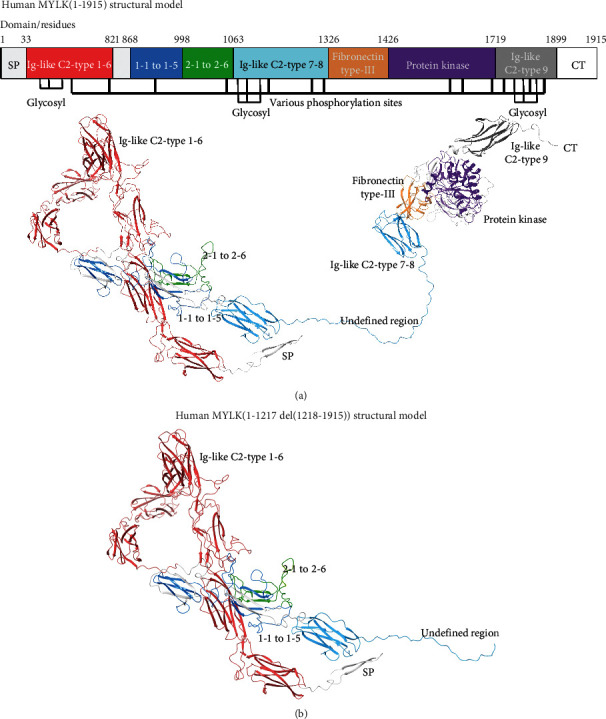
MYLK molecular model for the full-length human sequence consisting of 1914 amino acids and the variant p.S1218del. All protein residues shown are rendered in ribbons and colored by the domain map at the top. (a) Full-length model for the entire MYLK structure in ribbon is given and labeled by the domains in the key. (b) The p.S1218del structure for MYLK is shown and rendered similarly to that in panel (a). Colors in the domain legend key (top) match ribbon colors for both models shown.

**Figure 3:**
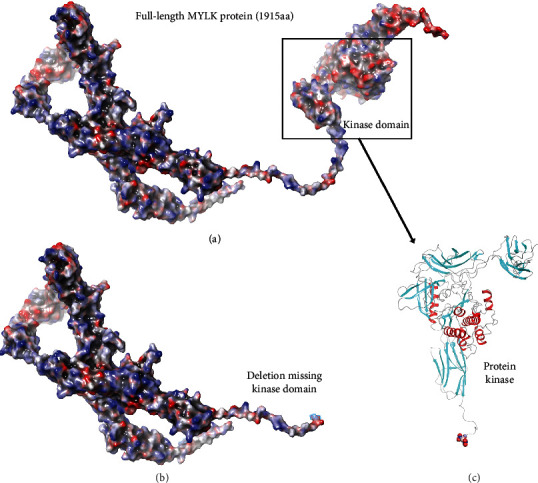
MYLK electrostatic mapping for full length and truncation. (a) Full-length model for the entire MYLK structure with electrostatics calculated using Poisson–Boltzmann (PB) calculation overlaid onto the structure. The kinase domain is boxed to emphasize its position in the protein. (b) Variant p.S1218del MYLK model is given with electrostatics overlaid indicating changes in charge. Here, the deletion of the crucial kinase domain is shown. (c) As indicated in the boxed region, the secondary structure for the protein kinase domain region is shown (rotated 90° to emphasize the catalytic region (on top).

For the wild type versus the variant p.S1218del, there is a clear loss of the kinase domain, which would have loss of function effect on the protein. Additional domain regions missing in the variant include Ig-like C2-type 8, fibronectin type-III, and Ig-like C2-type 9 domains. Taken together, this would constitute a negative variant as the large *N*-terminus may participate in binding and cause additional loss of function that a heteromeric individual's wild-type MYLK would be competing for binding [15, 16, 21, 23, 25–27] (Figure 1); our modeling has previously supported such findings [13–21, 23–25, 28]. Additionally, significant loss of activity was seen due to the enzymatic region being cleaved from the protein's global structure (Figures 2 and 3).

## 3. Discussion

Pathogenic variants in *ACTA2* and *MYH11* have been described in families with adult-onset thoracic aortic disease. Zhu et al. reported two families with mutations in *MYH11* who presented with TAAD and patent ductus arteriosus. This report was the first to suggest an association between aortic disease and mutations affecting proteins, which are part of the SMC contractile apparatus [29]. Another study showed that pathogenic variants in *ACTA2* are responsible for 14% of familial TAAD confirming the significance of an intact SMC contractile unit for maintaining the structural integrity of the aorta [2].

Our proband was found to have a deletion of 167 kb within cytogenetic band 3q21.1. This deleted section was noted to contain a portion of *MYLK* (NM_053025.3), specifically exons 21–34, and a portion of the *PTPLB* gene. *PTPLB*, also known as *HACD2*, encodes for very-long-chain (3R)-3-hydroxyacyl-CoA dehydratase 2 and has not been associated with a known clinical disorder at present [30]. *MYLK* genetic gross deletions appear to be especially rare [31]. This deletion results in protein truncation and loss of the calmodulin-binding domain, which would be expected to disrupt the SMC contractile unit. In addition, this section of DNA is not expected to present with variable copy numbers in the general population [32]. According to the inclusion criteria set by the American College of Medical Genetics and Genomics (ACMG), the deletion of exons 21–34 in MYLK is rated as “likely pathogenic” by 1PVS1_strong (http://autopvs1.genetics.bgi.com/cnv/3-123332644-123386568-DEL) + 1PM2. Therefore, the genetic testing laboratory considered this a likely pathogenic deletion. Moreover, our structural modeling findings clearly indicate a loss of function due to lack of any kinase domain.

Autosomal-dominant aortic dissection has been reported in association with variants that disrupt the calmodulin-binding domain, including those that prematurely truncate *MYLK* [33, 34]. Mouse models have supported this as well [35]. In Wang et al.'s study, two families with acute aortic dissection were described [34]. Specifically, *MYLK* variants and at least 1 deletion leading to haploinsufficiency have been associated with predisposition to aortic disease [36]. There has been at least one *MYLK* frameshift variant identified that was expected to cause both nonsense-mediated decay and haploinsufficiency [36].

Similarly, our proband presented with extensive aortic dissection with no history of aortic enlargement suggesting that an association between *MYLK* pathogenic variants and such a phenotype is not unlikely. The age of symptom onset can be variable, even within families, which makes it a challenge to determine the penetrance of *MYLK* pathogenic variants [34].

Familial TAAD is most commonly inherited in an autosomal-dominant manner, meaning there is a 50% chance of children who inherited the same genetic predisposition [37]. Penetrance is expected to be reduced. Specifically, pathogenic variants in the *MYLK* gene have been reported to be associated with lower penetrance, as seen in this family, and later onset than individuals with pathogenic variants in *TGFBR1* or *TGFBR2* [36]. The proband's mother was in her 80s and had no known history of aneurysm. First-degree relatives with the *MYLK* deletion were recommended to undergo aortic imaging to rule out asymptomatic disease [37]. Additionally, complex traits or environmental exposures may have an influence of penetrance/variable expressivity of this Mendelian trait, such as hypertension, autoimmune issues, or steroid use.

## 4. Conclusions

We highlight the clinical utility of genetic testing in patients with TAAD and how further cascade evaluation of their relatives, once a pathogenic variant is identified, is crucial and can potentially lessen associated morbidity and mortality [37]. The application of personalized molecular modeling for a protein informatics perspective not only gives us the detailed analysis derived from statistical mechanics calculations for high-precision all-atom effects that come from dynamic changes in the protein structure due to gene aberration but also the added benefit of deducing the likelihood of pathogenicity embedded within variants with unknown clinical significance through an exhaustive and detailing approach.

## Abbreviations

MYLK: Myosin light chain kinase
MCMD: Monte Carlo molecular dynamics
TAAD: Thoracic aortic aneurysm and dissection
HDCT: Heritable disorders of connective tissue.
